# A(a)LS: Ammonia-induced amyotrophic lateral sclerosis

**DOI:** 10.12688/f1000research.6364.1

**Published:** 2015-05-14

**Authors:** Bhavin Parekh

**Affiliations:** 1 Department of Biomedical Science, University of Sheffield, Sheffield, S10 2TN, UK

**Keywords:** amyotrophic lateral sclerosis, ammonia, neurodegenerative disorders

## Abstract

Amyotrophic lateral sclerosis (ALS) is a dreadful, devastating and incurable motor neuron disease. Aetiologically, it is a multigenic, multifactorial and multiorgan disease. Despite intense research, ALS pathology remains unexplained. Following extensive literature review, this paper posits a new integrative explanation. This framework proposes that ammonia neurotoxicity is a main player in ALS pathogenesis. According to this explanation, a combination of impaired ammonia removal— mainly because of impaired hepatic urea cycle dysfunction—and increased ammoniagenesis— mainly because of impaired glycolytic metabolism in fast twitch skeletal muscle—causes chronic hyperammonia in ALS. In the absence of neuroprotective calcium binding proteins (calbindin, calreticulin and parvalbumin), elevated ammonia—a neurotoxin—damages motor neurons. Ammonia-induced motor neuron damage occurs through multiple mechanisms such as macroautophagy-endolysosomal impairment, endoplasmic reticulum (ER) stress, CDK5 activation, oxidative/nitrosative stress, neuronal hyperexcitability and neuroinflammation. Furthermore, the regional pattern of calcium binding proteins’ loss, owing to either ER stress and/or impaired oxidative metabolism, determines clinical variability of ALS. Most importantly, this new framework can be generalised to explain other neurodegenerative disorders such as Huntington’s disease and Parkinsonism.

## Introduction

Amyotrophic lateral sclerosis (ALS) is the most feared, frequent, flummoxing and fatal motor neuron disease ^1– 3^. It is a biphasic disease which starts insidiously, later followed by relentless progression once symptomatic ^4^. Although rare, it is a grim and demeaning illness: it slowly cripples and confines its victims in their own body, ultimately killing them by breathing failure within 3–5 years after onset ^2, 5^. No cure exists ^1^. Ever since Charcot’s description of ALS (1869), a classical view defines ALS as an adult-onset neurodegenerative disease of upper and lower motor neurons ^6, 7^. However, ALS is clinically characterised by variability about the type and degree of motor neuron and non-motor neuron involvement ^8^.

ALS pathology involves an interaction of multiple genes and environmental factors. Indeed, ALS is mainly a polygenic disease (70%–90); and the heritable form (familial ALS) contributes to merely 30% of total ALS cases ^3, 9^. Remarkably, mutant C9ORF72, TARDBP, FUS, and SOD1 genes account for 70% of all familial ALS cases ^10^. Evidence shows that environmental factors such as intense physical activity, cigarette smoking, viral infections, and the ingestion of non-protein amino acids (i.e. β-N-methylamino-L-alanine) play a role in ALS ^5, 11, 12^.

## ALS aetiology: an enduring enigma

Despite nearly 150 years of research, ALS remains an enigma ^13^, although, at the cellular, molecular and metabolic levels, a staggering and ever expanding list of pathogenic mechanisms have been linked to ALS ^13, 14^. These include protein aggregation, mitochondrial dysfunction, oxidative/nitrosative stress, endoplasmic reticulum (ER) stress, axonal transport defects, glutamate excitotoxicity, impaired macroautophagy, impaired glycolysis, neuroinflammation, and glucose and fat metabolism impairments ^13– 19^. However, hitherto no hypothesis exist that effectively links all these mechanisms to a singular central cause ^3^. Hence, despite steadily accumulating knowledge about ALS, a key question still lingers: what cause ALS?

## ALS: a multi organ disease

Since ALS is manifestly a neurological disorder, researchers have long embraced an intuitive neurocentric view of ALS, assuming that intrinsic neuronal pathology causes ALS ^7, 20^. Against this view, however, growing evidence suggests that ALS pathology extends well beyond neuronal cells and involves multiple organs ^7, 14, 21^. Unsurprisingly, ALS is now deemed as a systems disease ^20, 22^. Not only that, evidence increasingly shows that primary pathological events, inherited or acquired, within these organs may act as distal cause of ALS ^14, 21, 22^. Such evidence is reviewed below.

## Role of skeletal muscle

ALS starts and spreads from skeletal muscle ^14, 23^. Indeed, some early symptoms of ALS involve the neuromuscular system: muscle atrophy, cachexia (wasting), weakness, and fasciculation (twitches) ^7, 11, 14, 19, 23^. In fact, cachexia reduces survival of ALS patients ^19^. Reinforcing such observations, data from animal models of ALS showed neuromuscular dysfunction precede motor neurons loss ^14^. For instance, Frey *et al.* showed selective loss of fast-fatigable neuromuscular synapses of SOD1G93A mice by 6 weeks of age, 2 month before symptomatic phase ^24^. Cogently, a study showed that the expression of mutant gene (SOD1G93A) exclusively in skeletal muscle of transgenic mice caused cachexia, neuromuscular denervation, paresis, and motor neuron degeneration ^25^.

Skeletal muscle possesses mainly two types of muscle fibres: fast twitch and slow twitch ^26^. Lately, evidence suggests that in ALS fast twitch muscle motor units are selectively damaged before overt symptoms, whereas slow twitch motor units show damage after overt symptoms ^27, 28^. For example, a set of studies showed a rapid motor unit loss during the presymptomatic phase (5 weeks of age) in fast but not slow-twitch muscles of the SOD1G93A mouse ^27^. Accordingly, fast twitch muscle appears to be more susceptible to damage in ALS patients ^28^. Therefore, fast twitch muscle pathology appears to be the distal cause of ALS.

Together, those findings have led to the “dying-back” hypothesis ^14^. This holds that ALS is a distal axonopathy in which pathological changes first arise distally at the neuromuscular junction and progress backward toward the spinal cord cell body ^14^. That said, however, recent and prior research mandates refinement of this hypothesis. Recently, experiments in the SOD1G93A mice showed independent and parallel degeneration of both upper and lower motor neurons at early stage, hinting at a common pathological mechanism ^29^. Consistent with this, recent neuroimaging studies showed early stage involvement of upper motor neuron (UMN) in ALS patients ^30^. In fact, Gower (1886), Charcot’s contemporary, suggested simultaneous and independent degeneration of upper and lower motor neurons in ALS ^31^. Thus, a common but hitherto unidentified pathological factor emanating from skeletal muscle appears to damages both upper and lower motor neurons.

## Liver: an emerging locus of ALS

Aside from skeletal muscle, mounting evidence suggests that liver dysfunction commonly occurs in ALS. Indeed, literature on the liver pathology in ALS has existed for over a half century ^32, 33^. Earlier, researchers showed a range of liver abnormalities in ALS patients including the disturbance of unconjugated bilirubin metabolism, mitochondrial defects, and copper accumulation in hepatic lysosomes ^32^. More recently, clinical studies suggest that hepatic steatosis (fatty liver degeneration) is a common and unique phenomenon in motor neuron diseases including ALS ^22, 34, 35^. Nodera *et al.* found that hepatic steatosis was present in 76% of ALS patients ^22^. In line with this, studies showed reduced growth hormone/insulin-like growth factor-1 (GH/IGF-I) levels, which induce hepatic steatosis, in ALS ^21, 36^. In keeping with this, hyperhomocysteinemia, which is associated with hepatic fat accumulation, commonly occurs in ALS patients ^37, 38^. Moreover, research showed that ALS-associated environmental factors such as virus infection (i.e. retrovirus virus and HIV) and cigarette smoking cause hepatic steatosis ^5, 12, 39, 40^. Furthermore, viral hepatitis, which causes hepatic insufficiency and frequent fatty liver degeneration, has been linked to motor neuron disease ^41– 44^. Finally, Reye-like syndrome, associated with fatty liver degeneration, has been associated with spinal muscular atrophy (SMA), a lower motor neuron disease ^35^.

A number of genetic findings also support this notion. Iron dysregulation disorders such as HFE gene-related hemochromatosis and hyperferritinemia, which induces hepatic steatosis, frequently (30%) occurs in ALS ^45, 46^. Additionally, mutant cholesterol and lipid pathways genes such as TDP-43 ATXN2, paraoxonase and CYP7A1, implicated in hepatic steatosis, have been linked to ALS ^47– 55^. Moreover, an interaction between disturbances in hepatic mitochondrial function and ER homeostasis causes hepatic steatosis; and investigators discovered morphological changes in ER structure and mitochondria in the liver of ALS patients ^32, 56^. These findings support the evidence that mutant ER-stress regulating genes such as XBP1, SigR1, VCP, TDP-43, FUS, SOD1, and VAPB are linked to ALS ^57, 58^. Furthermore, SMN gene, implicated in ALS and SMA, have been shown to regulate the development and function of liver ^35^.

Finally, hepatic steatosisis is linked to the metabolic syndrome, characterised by hyperglycaemia, hyperglucagonemia, insulin resistance and altered serum triglycerides; and such findings have been reported in ALS ^59– 65^. In this regard, it is interesting to note that damage to fast twitch skeletal muscle, the main site of glucose disposal and the largest reservoir of glycogen in humans, leads to hepatic steatosis ^66^.

Notably, Li *et al.* showed exendin-4, which counteracts hepatic steatosis, ameliorated motor neuron degeneration partly by correcting this systemic metabolic alteration ^67, 68^. This clearly suggests that, much like skeletal muscle, liver pathology is not merely an innocent bystander, but rather a premorbid condition, which plays an active role in ALS pathogenesis.

## Aims

Thus, (i) identifying skeletal–muscle produced unknown pathological factor, (ii) unravelling its nexus and synergism with hepatic steatosis, (iii) understanding the mechanisms by which this pathology factor causes motor neuron damage, and (iv) revealing the cause(s) of clinical heterogeneities would fully untie the Gordian knot of ALS pathology, allowing the development of predictive and prognostic biomarkers as well as potent drugs ^3, 13^. Hence, by taking a systems view, this paper aims to fill these knowledge gaps. Moreover, by fusing these separate pieces together, this paper presents a full picture of ALS pathology.

## Impaired glycolysis in fast twitch muscle: one of the pathological triggers of ALS

Evidence suggests that defective energy deficit in skeletal muscle triggers ALS. Investigators found impaired skeletal muscle metabolism, characterised by low ATP levels and hypermetabolism, causes neuromuscular dysfunction in ALS mouse model ^69, 70^. Conversely, metabolic interventions such as high-calorie diets and reducing hypermetabolism improved survival and alleviated symptoms in ALS ^19, 70^. However, the functional link between skeletal muscle metabolic impairment and ALS remains nebulous. Instructively, since ALS begins from fast twitch muscle, which relies on anaerobic glycolysis for energy (i.e., ATP), this immediately suggests that impaired anaerobic glycolysis produces the unknown pathological trigger.

## Impaired glycolysis in ALS

Compelling evidence suggests that muscle glycolysis is impaired in ALS. Valosin-containing protein (VCP), a gene linked to ALS, causes defective muscle glycolysis and reduced ATP levels. Dupis *et al.* linked upregulation of mitochondrial uncoupling proteins UCP1 and UCP3—which suppresses glycolysis and causes hypermetabolism—to muscle denervation in ALS ^71^. Bernardini *et al.* showed low expression of glycolysis genes such as FBP2 and enolase 3 in the skeletal muscles of ALS patients ^72^. Brockington *et al.* uncovered down regulation of glycolytic enzyme lactate dehydrogenase 1 in the VEGF ^δ/δ^ mouse model of ALS ^73^. Moreover, experiments showed that the gain-of-interaction of the SOD1G93A mutant with cytosolic malate dehydrogenase induces glycolytic impairments ^74^. Dunckley *et al.* linked variants of FLJ10986, a protein linked to glycolysis, with the susceptibility of sporadic ALS ^75^. Collectively, these findings clearly show impaired glycolysis in skeletal muscle of ALS patients and mouse model.

## Impaired muscle glycogen and glucose homeostasis in ALS

Notably, fast twitch skeletal muscle glycolysis depends on muscle glycogen storage and glucose transporter 4 (GLUT4)-mediated muscle glucose uptake ^26, 76^. Accumulating evidence suggests defective muscular glycogen metabolism and impaired GLUT4-mediated muscular glucose uptake in ALS. Derave *et al.* discovered diminished muscle ATP and glycogen accumulations in SOD1 G93A mice ^27^. Smittkamp *et al.* revealed impaired insulin-stimulated glucose uptake exclusively in fast twitch skeletal muscle in middle-stage SOD1 G93A mice ^77^. Accordingly, fast twitch skeletal muscle fibres of TDP-43 transgenic mice show defective insulin-induced GLUT4 translocation and glucose uptake ^77^. Moreover, in the mutant TDP-43-linked ALS mice, Perera *et al.* reported decreased AMPK, which mediates muscle contraction-induced glucose entry and glycogen synthesis ^76, 78, 79^. Conversely, AMPK activator drugs (i.e. latrepirdin) delayed ALS in SOD1G93A mice ^80^. Furthermore, muscle contraction facilitated glucose uptake involving Ca2+/calmodulin-dependent GLUT4 translocation appears to be defective in ALS. For example, investigators linked mutant neuregulin-ERBB4 gene, involved in calcium-induced glucose uptake during muscle contraction, to ALS ^79, 81^. Thus, it is obvious that ALS involves impaired carbohydrate metabolism that supports muscle glycolysis.

## ALS resistance of extraocular muscles (EOMs): role of glycolysis

Finally, the metabolic characteristics of—ALS-resistant—extraocular muscles (EOMs) further consolidate this notion ^82^. Two fundamental differences exist between EOMs and skeletal muscle metabolism ^83^. First, compared to skeletal muscles, EOMs have high glycolysis capacity, evident by the overexpression of glycolytic enzymes (e.g. lactate dehydrogenase, enolase) ^83^. Second, owing to their high vascularity, EOMs rely more on instantaneous glucose uptake—less on glycogen storage and GLUT4-mediated muscle glucose uptake ^83^. All in all, these three sets of findings point that defective glycolysis causes ATP deficits in fast twitch skeletal muscle of ALS patients. Hence, the unknown pathological factor emanating from skeletal muscle appears to have direct connection with defective muscle glycolysis. How?

## Ammonia: the elusive pathological factor

**Figure 1.**
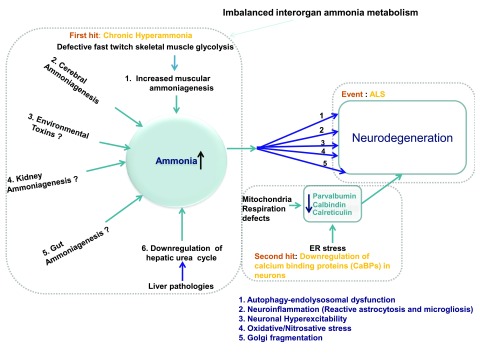
Line diagram: mechanisms of motor neuron damage in ALS. Mechanism of motor neuron degeneration in ALS involves two main factors: (i) ammonia neurotoxicity and (ii) down regulation of neuronal calcium binding proteins (CaBPs). Owing to imbalanced interorgan ammonia metabolism, ammonia, a well-known neurotoxin, accumulates in neurons. Among the five organs (brain, skeletal muscle, gut, liver and kidney) involved in ammonia metabolism, ALS appears to mainly involve the role of liver and skeletal muscle in that confluence of impaired ammonia removal—owing to impaired hepatic urea cycle—and increased muscular ammoniagenesis—owing to impaired glycolysis in fast twitch skeletal muscle—lead to chronic hyperammonia in ALS. In the brain, ammonia activates several neurodegenerative pathways such as (1) autophagy-endolysosomal dysfunction (2) neuroinflammation (3) oxidative stress (4) Golgi fragmentation and (5) neuronal hyperexcitability. In the absence of neuronal calcium binding proteins (CaBPs) such as parvalbumin, calbindin, calreticulin, activation of these degenerative pathways lead to motor neuron damage. Notably, decrease in calreticulin, because of increased ER stress, leads to lower motor neuron damage, whereas the down-regulation of parvalbumin and calbindin, because of defective mitochondrial respiration, leads to upper motor neuron damage.

**Figure 2.**
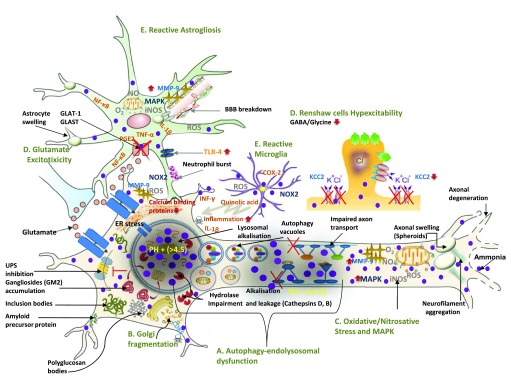
A molecular model of ammonia-induced motor neuron degeneration in ALS. (Modified with permission from 232). Ammonia intoxication directly damages motor neurons through five mutifactorial pathological mechanisms: 1) alkalisation-induced impairment of macroautophagy-endolysosomal system, 2) Golgi impairment, 3) increased oxidative/nitrosative stress and MAPK up-regulation 4) neuronal hyperexcitability and 5) neuroinflammation. These mechanisms explain frequently found cellular, molecular and neurophysiological phenotypes of motor neuron damage in ALS. **A**. Owing to ammonia-induced alkalisation, impairment of macroautophagy-endolysosomal system induces several key molecular histopathological features of ALS including : (i) ubiquitinated (Lewy and skein body-like inclusions) and non-ubiquitinated inclusion bodies (i.e. bunia bodies) formation, (ii) amyloid precursor protein (APP), (iii) gangliosides accumulation (i.e. GM2), (iv) autophagy vacuoles, (v) neurofilament aggregation and axonal swelling. **B**. Ammonia activates CDK5 which in turn leads to frequently observed Golgi fragmentation. **C**. Ammonia-induced oxidative/nitrosative stress and MAPK-up-regulation lead to multiple cellular and molecular pathological features such as: (i) blood brain barrier (BBB) breakdown, and (ii) MMP-9-induced ER stress. **D**. Ammonia causes neuronal hyperexcitability by (i) down-regulating astrocyte glutamate transporters (GLAT-1 and GLAST) and (ii) lowering potassium-chloride co-transporter KCC2 level which suppresses GABA and Glycine-mediated inhibitory neurotransmission. **E**. Ammonia leads to neuroinflammation secondary to reactive microglial and astrogliosis. This occurs because of (i) quinolic acid release from microglia (ii) up-regulation of pro-inflammatory cytokines (TNF, IL-1β, NF-κB, and PGE2) in astrocytes (iii) TLR-4 activation and (iv) neutrophil burst derived NADPH oxidase (NOX)-induced oxidative stress.

Notably, defective glycolysis, which reduces ATP levels, in fast twitch skeletal muscle activates catabolic reactions of adenine nucleotides (i.e. purine nucleotide cycle) and amino acids ( Figure 1) ^84, 85^. Intriguingly, such catabolic reactions produce ammonia—a neurotoxin 1000 times more toxic than ethanol at equimolar concentrations ^85, 86^. Since ammonia is toxic, it is obligatorily removed mainly through hepatic urea cycle which transforms ammonia into urea ^87^. Notably, when the urea cycle is impaired, as it occurs in fatty liver disease, increased ammonia production from skeletal muscle or from dietary sources can cause chronic hyperammonia (>35–50 µM) and consequent neurodegeneration and motor impairments ( Figure 1 and Figure 2) ^33, 88– 92^. Indeed, in many liver diseases, including fatty liver disease which commonly occurs in ALS, because of impaired urea cycle-mediated ammonia removal, hyperammonia frequently leads to corticospinal hyperexcitability, myelopathy and spasticity—features strikingly reminiscent of neurophysiological phenotypes of ALS symptoms ^93– 97^.

## Ammonia neurotoxicity: hypothesis and evidence

Together, these findings provide a compelling rationale for a new hypothesis. ALS pathology might involve not only skeletal muscle-induced increased ammonia production, because of impaired glycolysis, but also impair ammonia removal, secondary to hepatic steatosis-induced faulty urea cycle, leading to chronic hyperammonia and consequent progressive motor neuron degeneration ( Figure 1 and Figure 2). Astonishingly ammonia’s role has seldom been directly investigated. Nonetheless, diverse data obtained from clinical and animal studies support this hypothesis showing that hyperammonia increases ammoniagenesis and decreases ammonia removal in ALS.

A clinical study showed elevated ammonia level in motor neuron disease patients—with ammonia levels inversely correlated to disease duration ^98^. These investigators also found a causal relationship between ammonia and ALS by noting that infusion of amino acids, which causes ammoniagenesis, aggravates ALS ^98^. Moreover, dietary supplements of branched chain amino acids was one of the factors associated with the early onset of ALS (45 years) in Italian soccer players ^99^. Accordingly, other investigators reported accelerated skeletal muscle protein catabolism ALS ^100^. This chimes with the fact that hepatic steatosis-induced glucagon secretion, which occurs in ALS, increases ammoniagenesis through protein degradation ^101, 102^. Consistent with this, as noted above, intense or prolonged physical exertion, an ammoniogenic activity, is an ALS risk factor ^11, 103^.

Beside a link between hepatic steatosis and a faulty urea cycle, other lines of clinical evidence further implicate the impaired urea cycle in ALS ^48, 91, 104^. For example, Iłzecka *et al.* showed decreased arginine levels, an amino acid required for liver urea cycle function, in ALS patients ^105^. Additionally, research showed that metabolic acidosis, which impairs urea cycle, occurs in ALS ^60, 106^. Impaired hepatic urea cycle activates glutamine synthetase, an alternative ammonia detoxification pathway, and researcher also found increase in glutamine synthetase expression in blood platelets of ALS patients ^107, 108^.

Animal models of ALS further cement this ammonia hypothesis. Investigators showed hyperammonia and impaired urea cycle in 50 day old SOD1G93A mice compared to wild type mice of the same age ^109^. Moreover, these investigators showed increased glutamine, a precursor of ammonia, in SOD1G93A mice ^109^. Additionally, in the mutant SOD 1 G86R mice, de Aguilar *et al.* showed early (3 months of age) muscle denervation along with increased AMP deaminase-3 (AMPD3), an enzyme of purine nucleotide cycle involved in ammoniagenesis ^110^. Furthermore, a set of studies showed increased arginine vasopressin release in the SOD1 mice, and independent research showed that arginine vasopressin causes muscle protein degradation and consequent ammoniagenesis ^111, 112^. Conversely, research showed that ammonia-counteracting compounds such as phenylbutyrate, ariginine, resveratrol and l-carnitine alleviated symptoms and enhanced survival in the ALS mouse model ^97, 113– 118^.

Further supporting this hypothesis, experiments have shown that environmental neurotoxins implicated in ALS causes ammonia toxicity. Dietary intake of β-N-methylamino-L-alanine, a non-protein amino acid linked to Guam's ALS-PDS complex epidemic, causes liver damage and ammonia toxicity ^119, 120^. Similarly, the ingestion of *Lathyrus sativus* seeds, implicated in neurolathyrism (an upper motor neuron disease), causes liver dysfunction, urea cycle impairment, and chronic ammonia toxicity ^121, 122^. Finally, animal studies showed that the pesticide pyrethroid, which causes an ALS-mimicking syndrome, leads to protein catabolism ammonia toxicity ^123, 124^.

Yet another line of evidence bolsters the ammonia neurotoxicity hypothesis. Interestingly, reports showed that motor neuron disease could be one of Huntington's disease (HD)’s presenting features ^125, 126^. In fact, aside from genetic overlap with ALS, HD shares many pathophysiological characteristics with ALS: skeletal muscle atrophy, hepatic steatosis, hyperglycaemia and adipose tissue dysfunction ^127, 128^. Tellingly, although often regarded as curious findings rather than telltale observation, impaired urea cycle as well as hyperammonia occur in HD ^127^. Strikingly, data from mouse models of HD showed that protein-restricted diets not only reduced hyperammonia but also prevented the motor deterioration ^90^. This suggests that ammonia could be a common culprit in range of neurodegenerative conditions, especially affecting motor system.

In addition to muscles and the liver, ammonia metabolism involves other organs, including the gut, the kidneys and the brain ( Figure 1) ^87, 89^. Hence, this hypothesis does not preclude a role of these organs. Although no evidence has yet emerged to implicate the gut and kidneys in ALS, some data at least suggest a role of cerebral ammoniagenesis in ALS. Studies showed increased deamination of catecholamine, which causes cerebral ammoniagenesis, in ALS, evident by the overactivity of catecholamine oxidising enzymes such as MAO-B and aldehyde oxidase ^89, 129– 131^. Put together, these findings implicate ammonia neurotoxicty in ALS.

## Mechanisms of ammonia’s neurotoxicity

When hyperammonia occurs, ammonia enters into the brain, leading to neurotoxicity. Ammonia exerts pleiotropic neurotoxic effects by activating an array of cellular mechanisms, which are the proximal causes of ALS ( Figure 1 and Figure 2). These mechanisms include: 1) alkalisation-induced impairment of macroautophagy-endolysosomal system, 2) Golgi impairment, 3) increased oxidative/nitrosative stress and mitogen-activated protein kinase (MAPK) up-regulation 4) neuronal hyperexcitability and 5) neuroinflammation ^89, 132– 136^.

As described below, taken together, these five mechanisms not only explain several frequent cellular and molecular histopathological hallmarks of ALS but also neurophysiological features of ALS ( Figure 1 and Figure 2). The cellular histological features, explained by ammonia’s toxicity, include axon swelling, blood brain barrier breakdown and astrogliosis and microgliosis ^137– 139^. The molecular pathological features, explained by ammonia’s toxicity, include formation of inclusion bodies such as bunia bodies and Lewy bodies, gangliosides accumulation, glycogen aggregation, neurofilament derangement, Golgi fragmentation, and reduced glutamate transporters ^60, 140– 145^. Moreover, ammonia toxicity explains a key neurophysiological feature of ALS: neuronal hyperexcitability ^17^. Finally and most importantly, ammonia toxicity explains why ALS is mainly a motor neuron disease.

## Alkalisation-induced impaired macroautophagy-endolysosomal system

Ammonia-induced alkalisation impairs the macroautophagy-endolysosomal system, one of the main cellular garbage disposal systems. This occurs at least in two ways. First, ammonia, a weak base, preferentially accumulates in lysosomes because of their low acidity (PH~4.5) ^134^. Consequently, intra-lysosomal alkalisation and lysosomal enzyme leakages occur, impairing the lysosomal hydrolysis of proteins, lipids and carbohydrates ( Figure 2) ^134^. Second, ammonia alkalises acidic membranous compartments of axon terminals, jamming membrane microtubules and thereby blocking the anterograde-to-retrograde transport of endosomes ^146^. Consequently, impaired fusion of endocytic compartments with lysosomes occurs, causing defective autophagy of endocytosed material ( Figure 2) ^89, 146, 147^. As a result, toxic accumulation of protein aggregates, glycolipids, and carbohydrates occurs ^89^. In turn, these toxic by-products activate the apoptosis programme, causing cell death ^148^.

Ammonia’s alkalisation-induced toxicity is especially relevant to ALS because macroautophagy-endolysosomal dysfunction causes motor neuron degeneration ^149^. Indeed, mutant genes of this pathway such as SOD1, FIG4, CHMP2B, SQSTM1, DCTN1, DYNC1H1, and RAB7A have been linked to ALS ^149^. Consistent with this interpretation, research showed impaired dynein-dependent retrograde axonal transport, required for autophagosome-lysosome fusion, causes motor neuron degeneration ^150, 151^. Furthermore, consistent with lysosomal enzyme leakage, investigators reported increased lysosomal enzyme levels (i.e. acid phosphatase, Cystatin C) in the cerebrospinal fluid (CSF) and plasma of ALS patients ^152, 153^.

## Impaired lysosomal proteolysis

Ammonia-induced impaired lysosomal proteolysis explains key histopathological hallmarks of ALS including formation of inclusion bodies ( Figure 2). Ammonia-induced alkalinisation in lysosomes impairs the activities of protease enzymes including cathepsin B and cathepsin D ^134, 154^. Strikingly, investigators showed downregulation of cathepsin B and cathepsin D in ALS ^155, 156^. Notably, defective lysosomal proteolysis causes swollen axonal dystrophy (spheroids), with histological features such as ubiquitinated and non-ubiquitinated inclusion bodies, amyloid precursor protein, and neurofilament aggregation ^157^. In keeping with this, research revealed such findings in ALS ^144, 145, 156, 158– 161^.

In regard to non-ubiquitinated inclusion bodies, Kikuchi *et al.* showed that decreased cathepsin B generates Bunina bodies (small eosinophilic intraneuronal lysosomal inclusion bodies) in motor neurons, a hallmark of ALS ( Figure 2) ^140, 156^. Since cathepsin D mediates lipofuscin and α-synuclein clearance, and since downregulation of cathepsin D occurs in ALS, this explains frequently observed deposits of lipofuscin granules and α-synuclein aggregation in ALS patients ^148, 162– 164^. Moreover, reduced cathepsin B activity induces amyloid precursor protein (APP) accumulation, and Bryson *et al.* showed increased APP level in the SOD1 G93A mouse, which contributed to motor neuron damage ^158, 165^. As for impaired proteolysis-induced ubiquitinated inclusion bodies, ubiquitin inclusion aggregates such as Lewy body-like inclusions’ and ‘skein-like inclusions’ have been found in ALS ( Figure 2) ^145, 166^. This finding accords with the observations that inhibition of macroautophagy impairs the ubiquitin proteasome system (UPS) ^167^. Finally, neurofilament aggregation and spheroid formations have been found in the ALS mouse model and in patients ( Figure 2) ^161^.

## Impaired lysosomal ganglioside clearance

Gangliosides are complex sialylated glycosphingolipids, particularly found in the CNS ^168^. Notably, GM2 ganglioside is a main ganglioside in motor neurons ^169^. Accumulation of GM2 ganglioside, owing to impaired lysosomal Hexosaminidase (Hex) enzymes, frequently causes motor neuron disease ^170– 172^. For example, Banerjee *et al.* reported slow accumulation of GM2 ganglioside, primarily in motor neurons, in patients with progressive motor neuron disease associated with partial Hex A and no Hex B activity ^172^. By implication, this suggests that accumulation of gangliosides including that of GM2 occurs in ALS and that ammonia increases GM2 ganglioside levels. Indeed, although scantly investigated, some investigators reported increased ganglioside levels in ALS including GM2 ganglioside ^142, 173, 174^. In line with ammonia’s role in ganglioside metabolism, Perez *et al.* showed that ammonia causes leakage of Hexosaminidase A (Hex A), indicating GM2 accumulation ^175, 176^. Thus, ammonia-induced GM2 accumulation could partly explains the heightened vulnerability of motor neurons in ALS ( Figure 2).

## Impaired lysosomal carbohydrate clearance

Animal and clinical studies reported neuronal and glial glycogen accumulation and polyglucosan bodies (branched chained glycogen aggregates) in ALS ( Figure 2) ^60, 177, 178^. Notably, Dodge *et al.* showed that decreased level of α-glucosidase—a glycogen degrading lysosomal enzyme—partly causes glial and neuronal glycogen accumulation in ALS, and experiments showed that ammonia leaks α-glucosidase from lysosomes ^60, 179^. Thus, ammonia-mediated lysosomal dysfunction explains yet another histological feature of ALS. Of note, this fits with the observations that upper and lower motor neuron lesions frequently arise in polyglucosan body diseases ^180^.

## Impaired Golgi function

Ammonia toxicity could explain Golgi apparatus fragmentation in ALS, an early and frequently observed event ^141^. Sun *et al.* showed that CDK5 activation fragments Golgi apparatus ^181^. Interestingly, Cagnon and Braissant showed that ammonia activates CDK5. They also showed that CDK5 activation led to neuronal cell death and impairment of axonal outgrowth ^135^. Apparently, p25-induced mislocalization and deregulation of CDK5 activity occurs in ALS ( Figure 2) ^143, 182^. In fact, Nguyen *et al.* reported that an attempted re-entry of motor neurons into the G1-S phase of the cell cycle subsequent to CDK5 deregulation is a critical step of neurodegeneration in ALS ^182^.

## Increased oxidative/nitrosative stress and MAPK expression

Additionally, data suggested that ammonia induces oxidative/nitrosative stress and MAPK expression, frequently found pathological features of ALS ( Figure 2) ^132^. Research showed that oxidative/nitrosative stress and MAPK increases extracellular matrix degrading enzymes such as urokinase-type plasminogen activators and MMP-9 ^183^. Unsurprisingly, experiments found that increased levels of these extracellular matrix degrading enzymes occur in ALS ^184^. Strikingly, Kaplan *et al.* observed overexpression of MMP-9 increased the vulnerability of fast fatigable limb-innervating motor neuron ^185^. MMP-9 appears to exert neurotoxicity mainly through up-regulation of ER stress ( Figure 2) ^185^. Moreover, Skowrońska *et al.* showed that increase in MMP-9, which degrades the extracellular matrix, destroys the blood brain barrier (BBB) ^186^. Predictably, Nicaise *et al.* showed impaired blood-brain and blood-spinal cord barriers in mutant SOD1-linked ALS rodents ^138^. Additionally, since MAPK regulates cytoskeletal homeostasis, ammonia-induced MAPK activation explains why cytoskeleton abnormalities such as intermediate filaments accumulation occur in ALS ^160, 187^.

## Neuronal hyperexcitability

Furthermore ammonia intoxication explains neuronal hyperexcitability in ALS—a cardinal characteristic of ALS ^16^. By decreasing potassium-chloride cotransporter KCC2, located in the brain and spinal cord, ammonia increases chloride levels in neurons ( Figure 2) ^188^. Increased neuronal chloride levels in turn suppress GABA and Glycine-mediated inhibitory neurotransmission, causing neuronal hyperexcitability ( Figure 2) ^189^. In keeping with this, Fuchs *et al.* discovered decreased KCC2 expression in ALS-vulnerable motoneurons in spinal cord and hypoglossal nuclei of SOD1-G93A mice but not in EOMs ^190^. Concordantly, researchers reported spinal motor neuron hyperexcitability and degeneration in ALS patients ^191^. In fact, Hübner *et al.* showed that KCC2 knockout mice died after birth owing to motor deficits that caused respiratory failure, a feature similar to ALS ^189^.

Furthermore, ammonia causes glutamatergic excitotoxicty. By MAPK activation and increasing oxidative stress, ammonia decreases the glutamate transporter EAAT2 (GLT-1) and glutamate-aspartate transporter (GLAST) (EAAT-1) in astrocytes ( Figure 2) ^133, 192^. Consequently, decreased transporters impair astrocyte-mediated high affinity glutamate uptake and clearance, leading to defective glutamatergic neurotransmission and excitotoxicity ^133, 192, 193^. In line with this, decreased GLT-1 and GLAST have been found in the spinal cord of SOD1 G93A mice and ALS patients ^194– 196^. Interestingly, increased CSF glutamate was associated with a spinal onset of the disease and with severity of the symptoms in 41% of ALS patients ^196^.

## Neuroinflammation

Ammonia extensively affects the function of astrocytes and microglia ( Figure 2). Through several mechanisms including (1) quinolinic acid (QUIN) production, (2) NADPH oxidase (NOX) activity-induced reactive oxygen species (ROS) generation, (3) Toll-like receptor 4 (TLR-4) activation, and (4) extracellular-signal-regulated kinase (ERK) pathway stimulation, ammonia induces a transition from a resting state into reactive astroglia and microglia phenotype ( Figure 2) ^113, 197– 199^. Consequently, reactive astroglia and microglia increase oxidative stress and stimulate the release of a range of proinflammatory cytokines including NF-κB, IL-1β, and PGE2, leading to neuroinflammation and degeneration ( Figure 2) ^200^.

Emerging data indicate a role of QUIN in ALS ^201^. Chen *et al.* detected overproduction of serum tryptophan, kynurenine and QUIN in the CSF of ALS patients compared to controls, concomitant with microglial activation and neuroinflammation ( Figure 2) ^139^. Similarly, experiments showed increased microglial and neutrophil-derived NOX activity correlated with fast ALS progression ^202^. In keeping with increased ammonia-induced inflammation, investigators showed TLR-4 activation, and elevated levels of various pro-inflammatory cytokines in ALS ^203, 204^.

## Clinical heterogeneities in ALS: the role of calcium-binding proteins (CaBPs)

The postulated ammonia neurotoxicity as the sole cause of ALS raises an awkward question. If ammonia damages both upper and lower motor neurons equally, then why does ALS often deviate from its classical pattern, manifesting as either the upper or lower motor neuron dominant subtype ^8^? Moreover, why it is a relatively rare disorder? These questions clearly indicate that a protective factor exists that counteracts ammonia toxicity, and that anatomic-region specific loss of this factor causes the clinical heterogeneities in its presentation.

One such neuroprotective factor identified in ALS is the ER family of calcium binding proteins (CaBPs) ( Figure 1 and Figure 2) ^205^. By regulating voltage-gated calcium ion channels, CaBPs reduce calcium overload and cytotoxicity, thus protecting neurons from cell death ^205^. The CaBPs involved in motor neuron protection include calreticulin, parvalbumin, and calbindin which are distributed in anatomic region specific manner within motor neurons ^206, 207^. Calreticulin expression mainly occurs in limb-innervating lower motor neuron regions such as the lumbar spinal cord area and fast-fatigable motoneuron, whereas calbindin and parvalbumin are expressed in both lower and upper motor neurons ^206, 207^.

Differential anatomic region-specific distribution of CaBPs in the CNS partly explains different patterns of motor neurodegeneration ^206, 208^. In the SOD1 G93A ALS mouse model, during the presymptomatic stage, fast-fatigable motoneuron denervation mainly accompanies calreticulin loss ^208, 209^. By contrast, investigators showed that loss of calbindin and parvalbumin correlated with both upper and lower motor neuron damage ^206^.

## Region specific regulation of CaBPs: ER stress and bioenergetics

How do neurons lose different CaBPs in different anatomic regions of the CNS? Research showed that ER stress downregulates calreticulin in limb-innervating lower motor neurons motor. In fact, calreticulin co-localises with the ER ^207^. This accords with the finding that neuronal MMP-9—which enhances ER stress—selective damages fast fatigable lower motor neurons ^185^. Additionally, since androgens modulate ER stress, this explains why sexual dimorphism occurs in lower motor neuron damage ^210^. Interestingly, research revealed increased ER stress and reduced calreticulin in Alzheimer’s disease (AD) ^209^. This explains why AD occasionally co-exists with motor neuron disease ^211^.

As for the causes of reduced parvalbumin and calbindin expression in ALS, research implicates impaired oxidative metabolism secondary to defective mitochondrial electron transport (the respiratory chain) system ^212, 213^. Indeed, of the five protein complexes of the mitochondrial respiratory chain, research has frequently showed reduced respiratory chain complex I and IV activity in sporadic ALS patients ^205, 212^. Within these two complexes, complex IV appears to be particularly involved in ALS. This chimes well with the fact that 90% of all parvalbumin and calbindin-immunoreactive cells showed dense staining for respiratory complex IV (cytochrome c oxidase) ^214^. Furthermore, hyperhomocysteinaemia, found to be highly prevalent in ALS, damages mitochondria and suppresses respiratory complex IV activity ^37, 215^. Revealingly, compared to skeletal muscle, the EOMs have slow metabolism characterised by low complexes I and IV activities (~50%) yet elevated mitochondria density with increased complex I and IV levels (30% to 2 times)—explaining why parvalbumin and calbindin levels remain relatively unaffected in EOMs ^205, 216, 217^.

## The role of respiratory chain complex subunits

Interestingly, alterations in mitochondrial respiratory chain complex subunits also partly determine the spectrum of motor neuron damage. Investigators reported that altered Cytochrome c oxidase subunit Vb caused spinobulbar muscular atrophy, whereas Cytochrome c oxidase subunit I microdeletion induced upper motor dominant motor neuron damage ^218, 219^. Furthermore, deficiency of complex I involved lower motor neuron damage involving spinal and bulbar areas ^220^. This fits with the findings that anatomic region-specific differences in mitochondrial respiration contribute to the localized neurodegeneration ^221^.

In summary, these findings suggest that the regional loss of CaBPs expression, dependent on ER stress and defective mitochondrial respiration in the brain determines the anatomically variable manifestation of ALS. Collectively, it is also clear that motor neuron degeneration depends not only on postulated ammonia neurotoxicity but also on deficits of CaBPs within motor neuron.

## Biomarkers and therapeutics

From this insight about ALS pathogenesis, diagnostic, disease monitoring and therapeutic measures emerge—fostering real hopes that ALS can be halted or even cured. Because ammonia is a volatile organic compound, excreted from breath and skin, an ammonia breath test would present a simple, reliable, robust, inexpensive and non-invasive tool for diagnosis and monitoring of ALS ^222^. This ammonia breath test would prove invaluable in expediting drug discovery process. Aside from ammonia, gangliosides (e.g. Sialosylglobotetraosylceramide) and serum lysosomal enzymes could also serve as reliable adjuvant biomarkers of ALS ^142^.

As for therapeutics, since ammonia toxicity appears to be a major player in ALS, ammonia-removal strategies seem to be the most effective strategy for ALS treatment ^223^. Many existing ammonia-lowering agents including those that act on the hepatic urea cycle can be employed ^224, 225^. These could include salbutamol, conclevan, neomycin, sodium benzoate, ornithinephenyl acetate and L-ornithine aspartate ^223, 226, 227^. Moreover, since impaired fast-twitch skeletal muscle glycolysis plays a role in ALS, improving muscle glycolysis through various existing drugs such as serotonin agonists and AMPK agonists (e.g. D-xylose) is another promising pharmacological strategy ^228, 229^. Additional therapeutic strategies could involve correcting system metabolic defects such as hyperglucagonemia and acidosis ^60, 68^. Moreover, other potent therapeutic targets could involve MAPK inhibitors, K-Cl co-transporters, and hexosaminidase agonists (e.g. Pyrimethamine) ^192, 230, 231^. Finally, interventions that restore the levels of CaBPs should also be simultaneously applied for effective treatment.

## Summary

ALS is a ghastly and incurable disease. Despite increasing wealth of data, ALS remains poorly understood. By analysing existing literature, this paper has not only identified important knowledge gaps in ALS aetiopathology but also filled them and tied them together. In doing so, this paper postulates a new integrative explanation of ALS and suggests potent therapeutic measures to treat ALS. Central to this explanation is the notion that ALS is a neurological disease of metabolic origin—resembling hepatocerebral degeneration ^223^. This explanation posits that ALS pathology involves the interplay of two critical factors: 1) chronic hyperammonia caused by imbalanced interogan ammonia metabolism, mainly due to muscle and liver pathology ( Figure 1 and Figure 2) altered CaBPs homeostasis, mainly due to increased ER stress and impaired mitochondrial respiration ( Figure 1).

Considering all these together in sequence, impaired fast twitch skeletal muscle carbohydrate metabolism activates purinergic and amino acid catabolism, leading to a release of ammonia, a neurotoxin. Alternatively, ammonia toxicity can also be induced or exacerbated by other endogenous (e.g. cerebral deamination, intestinal ammoniagenesis) and exogenous sources (i.e. neurotoxins). Owing to concurrent liver pathology (e.g. hepatic steatosis) in ALS, impaired hepatic ammonia detoxification occurs. Consequently, ammonia levels progressively builds up, leading to chronic hyperammonia. Since ALS pathology also involves loss of neuroprotective CaBPs (i.e. calbindin, calreticulin and parvalbumin), ammonia neurotoxicity in the absence of CaBPs leads to ALS. Ammonia damages motor neurons through a range of pathways. These pathways include impaired macroautophagy-endolysosomal impairment, Golgi fragmentation, oxidative/nitrosative stress and reactive microglial and astrogliosis. These mechanisms explain a range of histopathological and neurophysiological hallmarks of ALS such as bunia bodies and neuronal hyperexcitability. Finally, since ALS appears to be associated with HD, dementia and Parkinsonism this framework can be generalised to explain these disorders ^33, 89, 90^.
