# Supplementary material for: A(a)LS: Ammonia-induced amyotrophic lateral sclerosis
Source: F1000Res. 2015 May 14;4:119. [Version 1] doi: 10.12688/f1000research.6364.1 (PMC5063041; doi:10.12688/f1000research.6364.1)
Supplement: Peer review history

# Peer review: Referee response for version 1

**DOI:** 10.5256/f1000research.6824.r18396

**Published:** 2016-12-12

**Authors:** Scott T Brady

**Affiliations:** 1 Department of Anatomy and Cell Biology, University of Illinois at Chicago, Chicago, IL, USA

ALS is a devastating disease of uncertain etiology and no effective therapies are available. As a result, there is a temptation to speculate about underlying causes in the hopes of hitting on the right answer. In this case, the author without any record of studying either ALS pathology or ammonia metabolism has proposed a novel integrative explanation in which impaired glycolytic metabolism in fast twitch skeletal muscle and liver pathology leads to chronically elevated ammonia levels that leads to altered Ca ^2+^ binding protein homeostasis due to ER stress and impaired mitochondrial respiration. Based on this idea, the author proposes that ammonia removal therapies would be effective treatments for ALS.

 The first requirement of any disease model is that it be consistent with known facts about the disease. Unfortunately, this model fails immediately on two counts. First, there is no evidence that interorgan ammonia levels are chronically elevated in ALS. People have been looking for plasma biomarkers for early diagnosis and none have reported elevated ammonia as a candidate in either ALS patients or animal models. The author does not cite any relevant patient studies and the one animal model cited (Bame, et al. 2014) does not find a significant correlation between ammonia levels and SOD1 G93A pathology. Similarly, liver pathology is not a hallmark of the disease. While skeletal muscle wasting is seen, this is associated with lack of activity, rather than a primary defect in glycolysis. 

                

 Second, ammonia toxicity is a well-documented condition in the brain and other tissues. While there are neurotoxic effects that include cortical atrophy, demyelination and edema (see for example Braissant, et al. 2013), none of these changes are specific to motor neurons (upper or lower) nor is there any evidence of ALS-like pathology in either patients with hyperammonemia or animal models with chronically elevated ammonia. Thus, patients with ALS show no evidence of elevated ammonia levels and patients with hyperammonemia do not have ALS-like symptoms. Curiously, both the Bame and the Braissant references are listed in the bibliography, but the conclusions are misrepresented as being consistent with the thesis. 

 Although the manuscript uses many buzzwords currently popular in the neurodegeneration field (autophagy, ER-stress, Ca ^2+^ homeostasis, neuroinflammation, etc.), the logic that relates changes in these parameters to ammonia metabolism is never made clear. The diagrams are convoluted and have little explanatory power. In particular, Figure 2 manages to be so densely packed with symbols and labels that it is uninterpretable. In contrast, the source figure from Kiernan, et al. 2011 that was adapted is sparse and focused.

 In sum, while the author has gathered a substantial bibliography, the main hypothesis is falsified by the literature and the utility of this opinion piece is therefore minimal. I would not consider this suitable for indexing in a rigorous journal.

I have read this submission. I believe that I have an appropriate level of expertise to state that I do not consider it to be of an acceptable scientific standard, for reasons outlined above.

# Peer review: Referee response for version 1

**DOI:** 10.5256/f1000research.6824.r16323

**Published:** 2016-10-07

**Authors:** Smita Saxena

**Affiliations:** 1 Institute of Cell Biology, University of Bern, Bern, Switzerland

This opinion article by Bhavin Parekh puts forward an interesting hypothesis about how ammonia neurotoxicity might influence and propagate ALS pathology. The article provides a concise introduction to the etiology of ALS with primary focus on the involvement of the skeletal muscle and liver in ALS pathogenesis. The author postulates a novel premise centering on the involvement of the liver and the distinctive occurrence of hepatic steatosis in motor neuron diseases including ALS. In depth literature is discussed for the existence of hepatic steatosis and hyperhomocysteinemia in ALS patients and other motor neuron diseases.

 The opinion article then focuses on ammonia and imbalances in interorgan ammonia metabolism and further discusses how this specific ammonia imbalance induces functional deficits in organelles (Golgi, ER, lysosomes), thereby impairing critical ALS associated pathways such as macroautophagy, oxidative/nitrosative stress, neuroinflammation, and hyperexcitability of motor neurons. Lastly, the authors search for a conceptual framework to account for clinical heterogeneity observed with respect to upper and lower motor neurons in ALS and they suggest that calcium binding proteins (CaBPs) are key molecules involved in neutralizing ammonia toxicity. The author further discusses the role of calcium binding proteins such as Calreticulin in ER stress and ALS pathogenesis and proposes that ammonia neurotoxicity and the parallel loss of expression of CaBPs leads to ALS.

 Overall the review is informative, well written and postulates an interesting hypothesis which might be of general interest to the neurodegeneration field. One of the limitations of this opinion article is that the discussion about CaBPs is restricted to Calreticulin in motor neurons, while recent studies have implicated various other ER chaperones in the ALS pathogenesis. The lack of these recent citations in the discussion concerning the involvement of ER chaperones in ALS does weaken the hypothesis. Nevertheless, this article provides an interesting area of reading and brings forward new experimental ideas for the further understanding of ALS and other neurodegenerative disorders.

I have read this submission. I believe that I have an appropriate level of expertise to confirm that it is of an acceptable scientific standard.

# Peer review: Referee response for version 1

**DOI:** 10.5256/f1000research.6824.r15775

**Published:** 2016-09-01

**Authors:** Viswanathan Krishnan, Joy Goto

**Affiliations:** 1 Department of Medical Pathology & Laboratory Medicine, University of California Davis School of Medicine, Davis, CA, USA; 2 California State University, Fresno, CA, USA

This Opinion Article introduces an interesting hypothesis connecting ammonia with ALS. The article initially begins with a discussion about the variety of etiologies of ALS and how their disparate onset and pathology is an unexplained area in the field. His main argument is the liver may be a locus for the variety of etiologies and specifically, hepatic steatosis is a unique link to motor neuron diseases, including ALS.

 This Opinion Article is replete with references supporting each of the statements from skeletal muscle to the various metabolic pathways (e.g. glycolysis and glycogen metabolism), to the deficits in vital organelles (e.g. lysosomes and Golgi). The latter part of the review introduces the role of calcium binding proteins and how ammonia dyshomeostasis contributes to neurodegeneration.

 The only shortcoming is the discussion on the mechanism of ammonia which mainly focuses on the *in vitro* application and exposure to ammonia; no chemical mechanisms following ammonia’s path of chemical reaction using labeled material is cited. While it is not the role of the Opinion Article to present these types of experiments. The lack of these cited references does weaken the hypothesis of ammonia’s connection to ALS. However, this article provides an interesting area of reading that may open new avenues of experimentation for researchers that are focused on understanding the connection between ammonia and not only ALS, but other diseases such as HD and PD.

We have read this submission. We believe that we have an appropriate level of expertise to confirm that it is of an acceptable scientific standard.
